# Bidirectional relationship between pain and sleep disturbance in middle-aged and older adults: evidence from the China health and retirement longitudinal study

**DOI:** 10.3389/fpsyt.2024.1485822

**Published:** 2024-11-28

**Authors:** Duan Yi, Mingyuan Yang, Qipeng Luo, Hong Li, Liang Kong, Qinghao Cheng

**Affiliations:** ^1^ Department of Pain Medicine, Peking University Third Hospital, Beijing, China; ^2^ Center of Anesthesiology and Pain, Emergency General Hospital, Beijing, China

**Keywords:** pain, sleep quality, sleep duration, China health and retirement longitudinal study, bidirectional correlation

## Abstract

**Background:**

Pain is one of the most prevalent symptoms that disrupt daily functioning and has been increasing in prevalence. Sleep disturbances frequently occur in individuals with pain, making it difficult to initiate and maintain sleep. This study aims to examine the bidirectional correlation between pain and sleep quality/duration among middle-aged and older Chinese adults

**Participants and setting:**

This study analyzed data from individuals aged 45 years and above who participated in both the 2018 and 2020 baseline surveys of China Health and Retirement Longitudinal Study (CHARLS).

**Methods:**

The bidirectional association between pain and sleep disturbance was assessed using multivariate logistic regression models, adjusting for various covariates.

**Results:**

Among individuals without pain, those with unsatisfactory sleep quality were more likely to experience future pain, with an adjusted odds ratio (OR) of 1.74 (95% CI: 1.57 - 1.92). Conversely, among individuals with satisfactory sleep quality, those with pain were more likely to develop unsatisfactory sleep quality in the future, with an adjusted OR of 1.87 (95% CI: 1.69 - 2.07). Additionally, shorter sleep duration (<6 hours) was significantly associated with pain status (OR=1.39; 95% CI: 1.28 - 1.50). The incidence of developing short sleep duration in individuals with pain was also higher (OR=1.49; 95% CI: 1.38 - 1.61).

**Conclusions:**

This research revealed a bidirectional relationship between pain and sleep disturbance in middle-aged and older Chinese adults, where each condition exacerbated the other. Recognizing and addressing this interconnected relationship was essential for effective management of both pain and sleep quality in this population.

## Introduction

Pain is one of the most prevalent symptoms that disrupt daily functioning, and its prevalence is steadily increasing. In China, approximately 30% of the population experiences pain (1, 2). Similarly, international studies show that pain conditions affect about 30% of American adults (3), 19% of European adults (4), around 18% of Australian adults (5), 12% of Spaniards, and 42% of Britons (6). These prevalence rates vary depending on specific non-malignant pain conditions, with higher rates observed particularly among women and older adults (7). Among Chinese individuals aged over 65 years, it is estimated that 80-85% suffer from pain-related ailments (8).

As people age, their physical and mental states undergo changes, and with the accelerating pace of modern life, an increasing number of middle-aged and elderly individuals are encountering sleep disturbance. Sleep disturbance is commonly observed in individuals with pain, creating significant challenges in both initiating and maintaining sleep amidst painful stimuli. The prevalence of sleep disturbance among those experiencing pain is alarmingly high, with reports indicating levels ranging from 50% to 70% even in clinical cohorts with milder pain conditions (9). Additionally, research has shown that the prevalence of sleep disturbance among individuals over 60 years old in China is 47.2% (10).

Although pain is often considered the primary cause of sleep disturbance, evidence from both animal and human studies suggests a more complex relationship. A bidirectional relationship exists, where pain disrupts sleep, and sleep deprivation or disorders, in turn, exacerbate pain (11–13). Over the past decade, numerous retrospective studies have examined the association between sleep and pain, emphasizing the need for longitudinal research to better understand their interplay (9, 14). Given the current aging population in China, there is a growing need tosl focus on elderly individuals who experience pain. However, most research on pain among elderly Chinese individuals has predominantly relied on cross-sectional surveys in recent years (15), with limited investigation into the progression of pain over time. This gap in evidence can be addressed through more rigorous longitudinal data collection and advancements in analytical methodologies (16).

Therefore, the objective of this study is to investigate the bidirectional correlation between pain and sleep quality/duration in elderly individuals using data from the China Health and Retirement Longitudinal Study (CHARLS).

## Methods

The data utilized in this study was derived from CHARLS, which employed a random sampling strategy to collect nationally representative, high-quality microdata from Chinese households. This is a national baseline survey, conducted every three years since 2011, encompassing a total of 450 communities (including villages) across 150 counties spanning over 28 provinces. The study included individuals aged 45 years and above who participated in both the CHARLS baseline surveys conducted in 2018 and 2020.

Participants who satisfied the following conditions were considered eligible for participation in this study: (1) comprehensive collection of demographic information, lifestyle factors, and health behavior data; (2) provision of responses regarding pain status, sleep quality, and sleep duration. Individuals were excluded if they lacked any of the relevant data mentioned above. Ultimately, 15,330 participants from the 2018 and 2020 waves of the survey were included to explore the correlation between pain and sleep quality/duration.

**Figure 1:**
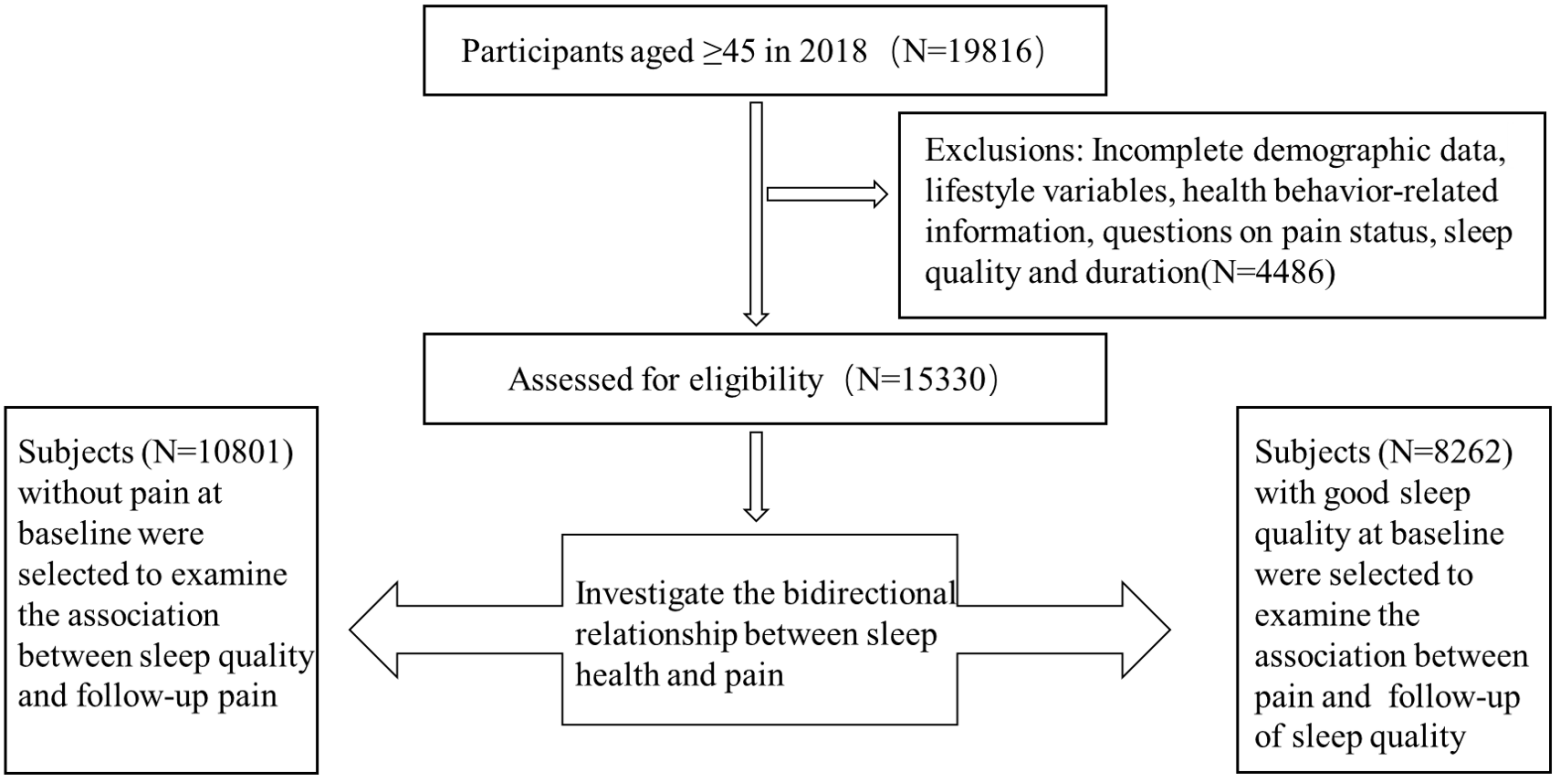
Flowchart of the study design.

In the first stage, participants who did not report any pain symptoms at the 2018 baseline were selected to examine the relationship between sleep quality/duration and the subsequent development of pain, resulting in a total of 10,801 eligible individuals. In the second stage, a subset of 8,262 participants with good baseline sleep quality was identified to investigate the relationship between pain and subsequent sleep quality/duration. The research received ethical approval from the Biomedical Ethics Committee at Peking University, with the fieldwork protocol also approved (Approval number: IRB00001052-11015). A detailed flowchart of the study design is presented in **Figure 1**

### Assessment of pain status

The definitions and manifestations of pain are diverse, and in this study. In this study, the pain issues addressed in the CHARLES questionnaire were precisely defined and utilized as the focus of research (17–19). According to the questionnaire, participants were inquired about their pain-related discomfort level: “ Are you often troubled with anybody pains? None? A little? Somewhat? Quite a bit? Very?” For this question, the term “pain” in this context encompasses the sensation experienced throughout various regions of the body.

### Assessment of the duration and quality of sleep

The diagnosis of sleep disturbance in this study was based on the CHARLES questionnaire, with a focus on the sleep-related issues encompassed within the questionnaire serving as the research subject matter. The evaluation of the duration and quality of sleep was based on question: “My sleep was restless.” and “During the past month, how many hours of actual sleep did you get at night?” The available choices for indicating one’s self-reported sleep quality encompassed rarely or none of the time (< 1day), some or a little of the time (1–2days), occasionally or a moderate amount of the time (3–4days), and most or all of the time (5–7days). The quality of sleep was categorized as either ‘satisfactory’ (occurring rarely or not at all, less than 1 day per week) or ‘unsatisfactory’ (occurring at least once a week). In accordance with previous research, nighttime duration of sleep was divided into three categories: insufficient (<6h), medium (6-9h), and sufficient (>9h).

### Covariates

The covariates analyzed in this research were categorized into demographic factors, data related to lifestyle variables and health behavior-related information. Demographic data include age, gender, educational level, marital status and residential regions. Lifestyle variables and health behavior-related information include labor force status, social interaction, alcohol consumption, smoking status, accident condition, and chronic disease history.

Age groups were categorized as follows: 45-64 years and 65 years or older. Educational level was categorized into four categories: Uncompleted primary education, primary education, secondary education and higher education. The marital status was categorized into two categories: other and married. Residential regions were categorized into rural and urban. The labor force status was classified into two categories: agricultural employment and non-agricultural employment or unemployment.

The social interaction was classified as either absent or occurring more than once a month. Regarding the consumption of alcohol, the term “drinking” was used to describe individuals who consumed beer, wine, or liquor at least once a month. Those who did not meet these criteria were categorized as “no drinking”. The term “smoking” was defined as engaging in the current or past behavior of using tobacco products. On the other hand, the term “no smoking” referred to individuals who did not engage in any form of tobacco use. Accident condition was classified into no accidents and previous accidents history (such as vehicular collision, accidental injury, history of falls or fractures). Chronic diseases were categorized as either having no or one concurrent chronic disease, or as having multiple comorbidities. Multimorbidity referred to the presence of two or more chronic diseases concurrently within an individual.

### Statistical analysis

Categorical variables were represented by frequencies and percentages, while continuous variables with a normal distribution were represented by means and medians. The Chi-square tests were utilized to compare the baseline characteristics. Binary logistic regression models were employed to analyze the association between baseline sleep quality/duration and subsequent pain status. Additionally, the study developed binary logistic regression models to determine whether baseline pain status could predict poor sleep quality. For sleep duration, multinomial regression models were employed to relationship the association between baseline pain and duration of sleep status. The findings were reported as odds ratios (ORs) accompanied by 95% confidence intervals (CIs). Statistical analysis was performed using SPSS software, and a significance P level of less than 0.05 was used to determine statistical significance.

## Results

### Baseline characteristics

#### Stage 1: association between baseline sleep quality and subsequent pain status

The baseline characteristics of individuals without pain are summarized in **Table 1**. A total of 10,801 participants were included in this stage of the study, with 5,713 (52.89%) reporting good sleep quality and 5088 (47.11%) reporting poor sleep quality. Individuals with satisfactory sleep quality were more likely to be men, have higher education levels, be married, lived in rural, engage in non-agricultural activities or be unemployed, be current or former smokers or drinkers, have no history of accidents, and be free from multimorbidity.

**Table 1:** Characteristics of participants at baseline in stages 1 and 2.

Characteristic	Sleep quality (N = 10801)		*P*	Pain status (N=8262)		*P*
	Good (N=5713)	Poor (N=5088)		No pain (N=5088)	Pain (N=3174)	
**Age, year**			0.003			<0.001
45–64	3755 (65.7%)	3482 (68.4%)		3482 (68.4%)	2001 (63.0%)	
≥ 65	1958 (34.3%)	1606 (31.6%)		1606 (31.6%)	1173 (37.0%)	
**Sex**			<0.001			<0.001
Male	3298 (57.7%)	2256 (44.3%)		2256 (44.3%)	992 (31.3%)	
Female	2415 (42.3%)	2832(55.7%)		2832(55.7%)	2182 (68.7%)	
**Educational level**			<0.001			<0.001
Uncompleted primary education	1832 (32.1%)	1834 (36.0%)		1834 (36.0%)	1572 (49.5%)	
Primary education	1925 (33.7%)	1552 (30.5%)		1552 (30.5%)	871 (27.4%)	
Secondary education	1189 (20.8%)	1101 (21.6%)		1101 (21.9%)	520 (16.4%)	
Higher education	767 (13.4%)	601 (11.8%)		601 (11.8%)	211 (6.6%)	
**Marital status**			<0.001			<0.001
Other	595 (10.4%)	656 (12.9%)		656 (12.9%)	505 (15.9%)	
Married	5118 (89.6%)	4432 (87.1%)		4432 (87.1%)	2669 (84.1%)	
**Residential regions**			0.0422			<0.001
Rural	2404 (42.1%)	2042 (40.1%)		2042 (40.1%)	959 (30.2%)	
Urban	3309 (57.9%)	3046(59.9%)		3046 (59.9%)	2215 (69.8%)	
**Labor force status**			<0.001			<0.001
Non-agricultural or no work	3760 (65.8%)	3192 (62.7%)		3192 (62.7%)	1813 (57.1%)	
Agricultural work	1953 (34.2%)	1896 (37.3%)		1896 (37.3%)	1361 (42.9%)	
**Social interaction**			0.71			<0.001
None	2963 (51.9%)	2658 (52.2%)		2658 (52.2%)	1870 (58.9%)	
More than once a month	2750 (48.1%)	2430 (47.8%)		2430 (47.8%)	1304 (41.1%)	
**Alcohol consumption**			<0.001			<0.001
No-drinking	3959 (69.3%)	3853 (75.7%)		3853 (75.7%)	2616 (82.4%)	
Current or former drinking	1754 (30.7%)	1235 (24.3%)		1235 (24.3%)	558 (17.6%)	
**Smoking status**			<0.001			<0.001
No-smoking	2928 (51.3%)	3088 (60.7%)		3088 (60.7%)	2130 (67.1%)	
Smoking	2785 (48.7%)	2000 (39.3%)		2000 (39.3%)	1044 (32.9%)	
**Accident condition**			<0.001			<0.001
No accidents	4964 (86.9%)	4165 (81.9%)		4165 (81.9%)	2212 (69.7%)	
Previous accidents history	749 (13.1%)	923 (18.1%)		923 (18.1%)	962 (30.3%)	
**Chronic disease**			<0.001			<0.001
No or one coexisting chronic disease	5138 (89.9%)	4310 (84.7%)		4310 (84.7%)	2363 (74.4%)	
Multimorbidity	575 (10.1%)	778 (15.3%)		778 (15.3%)	811 (25.6%)	
**Follow-up sleep quality**						<0.001
Good				1896 (37.3%)	730 (23.0%)	
Poor				3192 (62.7%)	2444 (77.0%)	
**Follow-up pain status**						
No pain	4943 (86.5%)	3964 (77.9%)	<0.001			
Pain	770 (13.5%)	1124 (22.1%)				

During the follow-up period, 770 (13.5%) participants with satisfactory sleep quality at baseline developed pain symptoms, compared to 1,124 (22.1%) participants with unsatisfactory sleep quality, who showed a significantly higher prevalence of future pain symptoms (P < 0.001).

#### Stage 2: association between baseline pain status and follow-up sleep quality

The baseline characteristics of individuals with satisfactory sleep quality are detailed in **Table 1**. In this stage, 8,262 participants were included, of whom 5,088 (61.58%) were pain-free, and 3174 (38.42%) reported experiencing pain. Factors associated with a higher likelihood of developing pain included advanced age, being female, lower educational attainment, unmarried status, residing in urban areas, working in agriculture, limited social interactions, non-smoking or non-drinking habits, history of accidents, and the presence of multiple health conditions.

During the follow-up period, 3192 (62.7%) individuals without pain continued to experience poor sleep quality, whereas the prevalence of poor sleep quality was significantly higher among those with pain (2444 participants, 77.0%). Individuals with pain were found to have a significantly increased risk of developing future unsatisfactory sleep quality compared to those without pain (P < 0.001).

### Prospective correlation between baseline sleep quality and pain status at follow-up

Among individuals without pain, those with unsatisfactory sleep quality at baseline were found to have a higher risk of developing pain, with a crude odds ratio (OR) of 1.82 (95% CI, 1.65–2.01). This association persisted after adjusting for covariates, with adjusted ORs of 1.74 (95% CI, 1.57–1.92), 1.73 (95% CI, 1.56–1.91), 1.72 (95% CI, 1.55–1.91), and 1.64 (95% CI, 1.48–1.82) across models 1, 2, 3, and the fully adjusted model 4, respectively (**Table 2**).

**Table 2:** Longitudinal association between sleep quality at baseline and risk of pain status (N = 10801).

Models for pain	Odds ratios of sleep quality at baseline (Ref. = good)	P value
Unadjusted	1.82 (1.65,2.01)	<0.001
Model 1	1.74 (1.57,1.92)	<0.001
Model 2	1.73 (1.56,1.91)	<0.001
Model 3	1.72 (1.55,1.91)	<0.001
Model 4	1.64 (1.48,1.82)	<0.001

Model 1 was adjusted for sex and age. Model 2 was adjusted for sex, age, education, residential region, and marital status. Model 3 was adjusted for sex, age, education, residential regions, marital status, alcohol consumption, smoking status, social interaction, and labor force status. Model 4 was adjusted for sex, age, education, residential regions, marital status, alcohol consumption, smoking status, social interaction, labor force status, accident and multimorbidity.

### Prospective correlation between baseline pain status and follow-up sleep quality

Among individuals with satisfactory sleep quality at baseline, those with pain were found to be at an increased risk of developing future unsatisfactory sleep quality, with a crude OR of 1.99 (95% CI, 1.80–2.20). This association remained significant after adjusting for covariates, with adjusted ORs of 1.87 (95% CI, 1.69–2.07), 1.91 (95% CI, 1.72–2.11), 1.91 (95% CI, 1.72–2.11), and 1.81 (95% CI, 1.63–2.01) in models 1, 2, 3, and the fully adjusted model 4, respectively (**Table 3**).

**Table 3:** Longitudinal association between pain status at baseline and sleep quality (N =8262).

Models for sleep quality	Odds ratios of Pain status (Ref. = no pain)	P value
Unadjusted	1.99 (1.8,2.2)	<0.001
Model 1	1.87 (1.69,2.07)	0.001
Model 2	1.91 (1.72,2.11)	0.001
Model 3	1.91 (1.72,2.11)	0.001
Model 4	1.81 (1.63,2.01)	0.001

Model 1 was adjusted for sex and age. Model 2 was adjusted for sex, age, education, residential region, and marital status. Model 3 was adjusted for sex, age, education, residential regions, marital status, alcohol consumption, smoking status, social interaction, and labor force status. Model 4 was adjusted for sex, age, education, residential regions, marital status, alcohol consumption, smoking status, social interaction, labor force status, accident and multimorbidity.

### Prospective correlation between baseline sleep duration and follow-up pain

Using individuals with a sleep duration of 6–9 hours per night as the reference group, it was found that shorter sleep duration was associated with an increased risk of developing pain. This effect persisted even after adjusting for covariates (OR = 1.39; 95% CI, 1.28–1.50). However, no statistically significant difference in future pain status was observed between individuals with sleep durations of 6–9 hours and those with sleep durations exceeding 9 hours (**Table 4**).

**Table 4:** Longitudinal association between sleep duration status at baseline and pain status (N = 15330).

Sleep duration status at baseline	Unadjusted		Model 1		Model 2		Model 3		Model 4	
	OR (95% CI)	P	OR (95% CI)	P	OR (95% CI)	P	OR (95% CI)	P	OR (95% CI)	P
< 6 h	1.96 (1.82,2.11)	<0.001	1.82 (1.68,1.96)	<0.001	1.77 (1.64,1.9)	<0.001	1.76 (1.63,1.89)	<0.001	1.39 (1.28,1.5)	<0.001
6-9h	Ref.	–	Ref.	–	Ref.	–	Ref.	–	Ref.	–
> 9 h	1.13 (0.95,1.36)	0.176	1.07 (0.89,1.28)	0.5	0.97 (0.81,1.17)	0.785	0.97 (0.8,1.17)	0.729	0.95 (0.78,1.16)	0.601

Model 1 was adjusted for sex and age. Model 2 was adjusted for sex, age, education, residential region, and marital status. Model 3 was adjusted for sex, age, education, residential regions, marital status, alcohol consumption, smoking status, social interaction, and labor force status. Model 4 was adjusted for sex, age, education, residential regions, marital status, alcohol consumption, smoking status, social interaction, labor force status, accident, multimorbidity and pain baseline status.

### Prospective correlation between baseline pain status and sleep duration at follow-up

The impact of baseline pain status on nighttime sleep duration is presented in **Table 5**. Individuals without pain served as the reference group. The analysis revealed a significantly higher incidence of short sleep duration (< 6 hours) in individuals with pain. After adjusting for covariates, this association remained significant (OR = 1.49; 95% CI, 1.38–1.61) (**Table 5**).

**Table 5:** Longitudinal association between pain status and sleep duration status (N = 15330).

Models for sleep duration status (N = 15330)	Sleep duration status					
	< 6 h		6-9 h		> 9 h	
	OR (95% CI)	P value	OR (95% CI)	P value	OR (95% CI)	P value
Unadjusted	2.06 (1.92,2.22)	< 0.001	Ref.	–	0.99 (0.80,1.22)	0.938
Model 1	1.92 (1.79,2.07)	< 0.001	Ref.	–	1.00 (0.81,1.23)	0.984
Model 2	1.87 (1.74,2.01)	< 0.001	Ref.	–	0.90 (0.72,1.11)	0.310
Model 3	1.85 (1.72,2.00)	< 0.001	Ref.	–	0.89 (0.72,1.10)	0.270
Model 4	1.49 (1.38,1.61)	< 0.001	Ref.	–	0.95 (0.76,1.18)	0.640

Model 1 was adjusted for sex and age. Model 2 was adjusted for sex, age, education, residential region, and marital status. Model 3 was adjusted for sex, age, education, residential regions, marital status, alcohol consumption, smoking status, social interaction, and labor force status. Model 4 was adjusted for sex, age, education, residential regions, marital status, alcohol consumption, smoking status, social interaction, labor force status, accident, multimorbidity and sleep quality baseline status.

## Discussion

Pain is a complex neurological response that leads to unpleasant sensations (20) and is widely recognized as one of the most common and disabling symptoms in medicine (21–23). Chronic somatic pain can progressively result in physical disability, depression, and sleep disturbance (20, 24, 25). This study identified a significant bidirectional association between sleep and pain, supported by a large sample size of longitudinal follow-up data. The findings remained consistent even after adjusting for baseline variables. These adjustments further reinforced our ability to reveal the intricate relationship between sleep disturbance and pain status.

Epidemiological studies had established that poor sleep was associated with a heightened susceptibility to experiencing pain (26). Persistent sleep disturbance and pain are believed to have a reciprocal relationship, where sleep disturbance heighten an individual’s susceptibility to pain (27). Consistent with our findings, there is a bidirectional correlation between the duration, frequency, and quality of sleep disturbance and the severity and location of pain (28). For instance, VINSTRUP et al. conducted a prospective cohort study involving 1,955 medical staff and observed a significant association between poor sleep quality and the incidence of back pain (29). Furthermore, studies have indicated a negative correlation between pain sites and sleep quality.

An increase in pain reports is often observed with a reduction in sleep duration (30, 31). In a study involving 1,773 adolescents, AUVINEN et al. identified insufficient sleep duration or poor sleep quality as independent risk factors for back pain (32). The duration and frequency of sleep disturbance show a significant association with somatic pain, with stronger effects observed in cases of prolonged duration and higher frequency of sleep disturbance (33). Sleep disturbance led to heightened sensitivity to pain and impact the brain’s ability to perceive pain thresholds and inhibit pain. Shortened sleep duration results in central sensitization of nociception and a decrease in pain regulation, thereby increasing the risk of pain. Additionally, prolonged wakefulness acts as a primary driver of hyperalgesia, significantly amplifying pain perception (34, 35). There is compelling evidence that sleep disruptions negatively affect the body’s natural pain inhibition mechanisms, leading to an escalation in spontaneous pain, particularly among women (36).

Preoperative insomnia had been demonstrated as a reliable predictor of postoperative pain, and addressing sleep disturbance can effectively mitigate postoperative pain (37). A meta-analysis also revealed that sleep disruptions prior to surgery adversely affect the onset and intensity of persistent pain among individuals recovering from surgery (38). Research conducted on healthy individuals revealed a positive correlation between sleep restriction and a higher occurrence of newly emerging spontaneous pain (39).

The correlation between sleep and pain is bidirectional: pain hinders sleep quality, while insufficient sleep or sleep disturbance exacerbate pain perception. The potential link between sleep disturbance and pain may be attributed to oxidative stress. Oxidative stress can be triggered in the brain experiencing sleep deprivation (35), and the various biomarkers indicative of oxidative stress and redox status exhibit significant circadian rhythms (40). A previous study indicated that sleep deprivation could exert a significant influence on pressure pain thresholds and plasma oxidative stress markers among healthy adult residents (41). Staying awake at night can elevate oxidative stress levels, while sleep acts as a protective measure against its harmful effects. Moreover, specific brain structures are responsible for encoding both pain and sleep. The nucleus accumbens (NAc) receives sensorimotor and sleep-related inputs (42–44), and subsequently transmits signals to multiple brain regions involved in the processing of pain perception and sleep, suggesting a bidirectional relationship between insufficient sleep and altered pain perception (45). The presence of sleep problems is also associated with the severity of pain, and sleep issues have been found to predict worsening pain over time, with this effect being more pronounced in women compared to men and potentially mediated by fatigue (46).

In the Chinese population aged between middle age and elderly, a significant association between body pain and falls had been documented (8), aligning with our study’s findings of a history of accidental trauma. A bidirectional relationship exists between sleep quality and pain intensity with age, where pain experienced during the day significantly impacts sleep quality at night, and insufficient or poor sleep quality at night results in heightened pain levels the following day (47). The prevalence of pain symptoms tends to increase with advancing age, although not universally among older adults. Pain in the elderly is often associated with the onset of chronic conditions, as the incidence of chronic diseases rises with age, potentially exacerbating pain. This study suggested that sleep quality moderated the relationship between the number of chronic diseases and the extent of pain experienced. Patients with chronic diseases often face challenges such as reduced sleep quality and difficulty initiating sleep.

At baseline, women, agricultural workers, unmarried individuals, patients with a history of trauma, and those with multiple chronic diseases exhibited a higher likelihood of experiencing sleep disturbance and pain. Research has indicated that females are more susceptible to pain due to factors such as reduced muscle mass and lower bone density compared to males, increased engagement in daily household chores, heightened sensitivity to pain stimuli, greater willingness to report pain symptoms, and poorer response to analgesics (48, 49). Additionally, the interplay between physiological and psychosocial mechanisms may factors could potentially play a role in explaining differences in how males and females perceive pain (50). Other studies had also reported an inverse relationship between education level/manual labor intensity and pain experience. Individuals with lower educational attainment or those engaged in agricultural manual labor tend to exhibit higher rates of pain, possibly due to frequent outdoor physical exertion (51, 52).

## Limitations

Firstly, the concept of pain encompasses a broad spectrum of experiences, encompassing both chronic and episodic pain, as well as physical and psychological discomfort. No division was made into types of pain in this study. Sleep disturbance was identified on CHARLS questionnaires, not by polysomnography. The reliance on self-reported data for pain and sleep may result in certain deviations. However, previous studies have demonstrated that self-reported multiple illnesses exhibit good specificity in large-scale population baseline studies (41). Future research should aim to conduct more comprehensive investigations from various perspectives to measure sleep and pain accurately. Secondly, due to missing data for some quality-of-life scores, correlational analyses were not feasible, limiting our ability to fully understand how other factors interact with sleep and pain. Additionally, despite the longitudinal analysis conducted in this study to further elucidate the association between sleep quality, duration, and pain, there is an urgent requirement for a more extensive comprehension of the underlying mechanisms linking sleep and pain intensity to optimize pain management interventions. Therefore, additional studies should be undertaken to thoroughly analyze and clarify the causal pathways connecting sleep and pain intensity. Finally, as the sample is drawn from the Chinese population, it might be worth mentioning any potential differences that might arise in different ethnic or geographical populations.

## Conclusion

The bidirectional correlation between poor sleep quality or insufficient sleep duration and pain in middle-aged and older Chinese adults was well established. We recommend focusing on sleep disturbance and pain in middle-aged and elderly patients while enhancing public awareness of sleep health and pain management. From a clinical perspective, this potential long-term bidirectional association emphasize the importance of evidence-based interventions targeting sleep and pain issues in older adults.

## Data Availability

The original contributions presented in the study are included in the article/supplementary material, further inquiries can be directed to the corresponding author/s.
